# Genomic predictions can accelerate selection for resistance against *Piscirickettsia salmonis* in Atlantic salmon (*Salmo salar*)

**DOI:** 10.1186/s12864-017-3487-y

**Published:** 2017-01-31

**Authors:** Rama Bangera, Katharina Correa, Jean P. Lhorente, René Figueroa, José M. Yáñez

**Affiliations:** 1 Aquainnovo S.A, Talca 60, Puerto Montt, Chile; 2 Facultad de Ciencias Veterinarias y Pecuarias, Universidad de Chile, Santa Rosa 11735, La Pintana, Santiago, Chile

**Keywords:** Genomic selection, Salmon Rickettsial Syndrome, Disease resistance, Reliability

## Abstract

**Background:**

Salmon Rickettsial Syndrome (SRS) caused by *Piscirickettsia salmonis* is a major disease affecting the Chilean salmon industry. Genomic selection (GS) is a method wherein genome-wide markers and phenotype information of full-sibs are used to predict genomic EBV (GEBV) of selection candidates and is expected to have increased accuracy and response to selection over traditional pedigree based Best Linear Unbiased Prediction (PBLUP). Widely used GS methods such as genomic BLUP (GBLUP), SNPBLUP, Bayes C and Bayesian Lasso may perform differently with respect to accuracy of GEBV prediction. Our aim was to compare the accuracy, in terms of reliability of genome-enabled prediction, from different GS methods with PBLUP for resistance to SRS in an Atlantic salmon breeding program. Number of days to death (DAYS), binary survival status (STATUS) phenotypes, and 50 K SNP array genotypes were obtained from 2601 smolts challenged with *P. salmonis.* The reliability of different GS methods at different SNP densities with and without pedigree were compared to PBLUP using a five-fold cross validation scheme.

**Results:**

Heritability estimated from GS methods was significantly higher than PBLUP. Pearson’s correlation between predicted GEBV from PBLUP and GS models ranged from 0.79 to 0.91 and 0.79–0.95 for DAYS and STATUS, respectively. The relative increase in reliability from different GS methods for DAYS and STATUS with 50 K SNP ranged from 8 to 25% and 27–30%, respectively. All GS methods outperformed PBLUP at all marker densities. DAYS and STATUS showed superior reliability over PBLUP even at the lowest marker density of 3 K and 500 SNP, respectively. 20 K SNP showed close to maximal reliability for both traits with little improvement using higher densities.

**Conclusions:**

These results indicate that genomic predictions can accelerate genetic progress for SRS resistance in Atlantic salmon and implementation of this approach will contribute to the control of SRS in Chile. We recommend GBLUP for routine GS evaluation because this method is computationally faster and the results are very similar with other GS methods. The use of lower density SNP or the combination of low density SNP and an imputation strategy may help to reduce genotyping costs without compromising gain in reliability.

**Electronic supplementary material:**

The online version of this article (doi:10.1186/s12864-017-3487-y) contains supplementary material, which is available to authorized users.

## Background

Salmon Rickettsial Syndrome (SRS) caused by an intracellular bacterium *Piscirickettsia salmonis* is considered one of the major diseases of the salmonid aquaculture industry in Chile [1]. SRS outbreaks can lead to severe economic losses to farmers because of the high mortality associated with the disease during salt water production [1, 2]. It has been estimated that in the Chilean Atlantic salmon (*Salmo salar*) industry, *P. salmonis* is responsible for up to 74% of infection-related mortality and economic losses of up to US$100 million. Antibiotic treatments may inhibit the growth of the pathogen, but have been unsuccessful in stopping disease outbreaks and pose serious health issues for fish and humans [3, 4]. Although there are more than 33 commercial vaccines available against *P. salmonis* they have not proven to be consistently effective under field conditions [1, 5].

Selective breeding for resistance against infectious diseases represents a realistic and sustainable approach to control disease outbreaks in livestock and aquaculture species [6, 7]. Traditional aquaculture selection programs for disease traits involves sib-testing where survival phenotype information comes from experimental infection of full-sib family groups of the selection candidates with a specific pathogen [8]. However, this method has limited reliability under classical selection schemes because breeding candidates are selected based on mid-parent (family) estimated breeding values (EBV) where only a maximum of 50% of the total genetic variation is exploited [9]. In addition, the use of only between-family variation to make selection decisions leads to increased co-selection among close relatives and imposes restrictions on inbreeding [9]. Nevertheless, previous studies in the same commercial Atlantic salmon population used in the present study estimated moderate to medium heritability (0.11 to 0.41) for resistance to *P. salmonis*, indicating the potential for selective breeding for *P. salmonis* resistance [10, 11].

Genetic markers associated with quantitative trait loci (QTL) alleles for disease resistance can be used in Marker Assisted Selection (MAS) of breeding candidates with genotypes, even in the absence of phenotypes, to accelerate genetic progress [12]. The carriers of favorable QTL alleles and its effects are usually identified through dense panels of Single Nucleotide Polymorphisms (SNP) using linkage and association mapping [12–14]. In Atlantic salmon, major QTLs explaining a considerable proportion of the genetic variation for resistance to infectious pancreatic necrosis [15, 16] have been successfully used for MAS in breeding companies [6, 17]. Recently, a genome-wide association study (GWAS) by Correa et al. [18] revealed that resistance to *P.salmonis* in Atlantic salmon is under moderate polygenic control. The same study identified five SNP significantly associated with *P.salmonis* resistance traits in chromosomes Ssa01 and Ssa17. However, due to the small amount of phenotypic variance explained by each marker, it was suggested that resistance to *P.salmonis* can be more efficiently improved with genetic evaluations incorporating dense SNP genotype information compared to MAS alone [18]. Genomic selection (GS) is an alternative method to MAS where information from genome-wide marker genotypes (e.g., SNP) are used in genetic evaluations so that all QTL are in linkage disequilibrium (LD) with at least one marker and selection is based on predicted genomic EBV (GEBV) [19–21]. In GS, sibs of the selection candidates with both phenotype and genotype are used to estimate each marker effect and are later used to predict GEBV for the selection candidates using only genotypic information [20]. In aquaculture, studies using simulated [22–25] and real data [9, 26] have shown the superior performance of GS methods in terms of increased genetic gain, accuracy of selection and lower rate of inbreeding.

Several GS methodologies varying with respect to assumptions about marker effects have been proposed for the genome-enabled prediction of EBV [27, 28]. The most widely used GS methods are the genomic best-linear unbiased prediction (GBLUP) approach using realized genomic relationship matrix calculated from the dense genome-wide SNP markers and Bayesian methods (e.g., Bayes A, Bayes B, Bayes C and Bayesian LASSO) [20, 28–30]. The performance of each of these GS methods varies according to the true underlying genetic architecture among traits and model assumptions [20, 28, 30, 31]. Therefore, it is valuable to compare the performance of different GS methodologies using real data to identify the best methods, i.e., those which provide accurate GEBV predictions over normal pedigree based EBV predictions.

The objectives of this study were i) to compare the reliability of commonly used genomic prediction methods for genomic selection under various underlying genetic models and pedigree based BLUP for *P. salmonis* resistance traits in Atlantic salmon and ii) to evaluate the effect of different marker densities on the reliability of genomic predictions for different genomic prediction models and pedigree based BLUP for *P. salmonis* resistance traits in Atlantic salmon.

## Methods

### Fish material and challenge test

The breeding program for Atlantic salmon was started by the company AquaChile (Puerto Montt, Chile) in the year 1997 with the aim of improving economically important traits. The base population of the breeding nucleus originated from the Irish strain Fanad-Mowi (originally from Norway) [32, 33] and was introduced during the 1990s to Chile through commercial agreements. At present, the breeding program is managed by the breeding company Aquainnovo SA at Salmones Chaicas (Puerto Montt, Chile). All fish material used in this study was from the same breeding program and corresponds to the year-class 2010, which has undergone four generations of selection mainly for harvest weight in Chilean farming conditions. The fish were hatched during May 2010 with an approximate mating ratio of one sire to two dams in most cases. A total of 118 families were produced as progeny of 40 sires and 118 dams and reared in separate tanks until tagging. The required number of fish from all families were tagged individually at an average weight of 13.1 g (SD = 3.4 g) using Passive Integrated Transponder-tag (PIT-tag), in order to keep pedigree information. Tagged fish were reared in a single communal tank for about 14 months before transfer to the Aquainnovo’s Research Station located in Lenca River, X^th^ Region, Chile. After a 29 day acclimation period in salt water (31 ppt) a total of 2601 fish, an average of 22 (ranging between 9 and 24) fish per family, weighing on average 274.8 g (SD = 90.6 g) were experimentally challenged with *P.salmonis* as described previously [10, 18]. In brief, prior to the challenge test, the fish tested negative for the presence of Infectious Salmon Anaemia virus, Infectious Pancreatic Necrosis virus, *Renibacterium salmoninarum* by RT-PCR and negative for *Flavobacterium spp.* culture. To induce infection, fish were injected with 0.2 ml of a LD50 inoculum of *P.salmonis* through intra-peritoneal (IP) injection. Post IP injection, infected fish were distributed equally by family into three different tanks with salt water (31 ppt) such that each of the 118 full-sib families were represented in all three tanks. The challenge test continued for 40 days and mortalities were recorded daily. The Kaplan-Meier curves of the survival function was plotted for the test period to show the cumulative mortality across the challenge (Additional file 1: Figure S2). All surviving fish at day 40 were anesthetized and euthanized. Tissue samples (fin clips) for genomic DNA isolation were taken from all fish and preserved in 95% ethanol at −80 °C. The procedures for challenge and sampling were approved by The Comite de Bioetica Animal, Facultad de Ciencias Veterinarias y Pecuarias, Universidad de Chile (Certificate N^0^ 08–2015).

### Genotype data

Genomic DNA was isolated from the stored fin clip samples of all 2601 challenge tested fish using a commercial kit (DNeasy Blood & Tissue Kit, Qiagen), following manufacturer’s protocol. Genotyping was performed using a 50 K Affymetrix® Axiom® myDesign™ SNP Genotyping array designed by the joint collaboration of AquaInnovo SA and the University of Chile. The 50 K SNP array used in this study was derived from a previously developed and validated custom made 200 K SNP array [34] based on several quality control criteria as described previously [18]. Importantly, the 50 K SNP array had markers distributed equally across the genome with a distance of more than 10Kb from its neighboring SNP [18]. Genotypes of all fish samples were obtained following Best Practices Analysis Workflow from Affymetrix [35] and selection of Poly-high-resolution and the No-minor-homozygote SNPs using SNPolisher [35]. To filter SNPs quality control of the SNP genotype data was performed based on the Hardy Weinberg equilibrium (*p* < 1 × 10^−10^), Minor Allele Frequency (>0.001) and the call rate for SNPs and samples (>0.95). The quality control step resulted in a total of 2392 individuals and 49,684 SNPs distributed across the genome for further analysis.

### Phenotypic records and trait definitions

Resistance to *P. salmonis* was considered to be challenge survival, defined as the time to death measured in days (DAYS) with values ranging from 1 to 40 depending on the day the fish died; and as binary survival status (STATUS), scored as 1 if the fish died during the 40-day challenge and 2 if the fish survived until the end of the challenge. Thus, the fish with higher DAYS and a STATUS of 2, were assumed to be more resistant animals. Test tank designation and final body weight on the day of death or at the end of the challenge for survivors were recorded.

### Breeding value estimation

The two resistance traits DAYS and STATUS were analyzed separately as a linear trait and threshold trait, respectively in univariate models. The EBV were estimated using polygenic pedigree based BLUP (PBLUP) [36]. The SNP effects and GEBV were estimated using polygenic pedigree and/or SNP genotype information on the basis of genomic BLUP (GBLUP) [36], SNPBLUP [37], Bayes C [38, 39] and Bayesian LASSO [30, 40].

#### Pedigree based BLUP

The conventional pedigree-based variance components and EBV were estimated using PBLUP:

M1 $$ \boldsymbol{y}=\boldsymbol{X}\boldsymbol{\beta } +\boldsymbol{T}\upmu +\boldsymbol{e} $$

where β is a vector of fixed overall mean and age of fish at challenge (AGE) as co-variate, $\boldsymbol{\upmu}$ is a vector of random additive genetic polygenic effects with a distribution $\sim \mathrm{N}\left(0,\boldsymbol{A}{\sigma}_u^2\right)$, $\boldsymbol{e}$ is the vector of random error effects with a distribution $\sim \mathrm{N}\left(0,\boldsymbol{I}{\sigma}_e^2\right)$, *X* and *T* are the incidence matrices, $\mathbf{A}$ is the pedigree-based additive genetic relationship matrix [41] and $\boldsymbol{I}$ is the identity matrix. The trait DAYS was analyzed as a linear trait using AIREMLF90 and GIBBS1F90, whereas, the trait STATUS was analyzed as a threshold-linear trait in THRGIBBS1F90 in BLUPF90 family programs [36]. Briefly, AIREMLF90 uses Average-Information REML for estimating variance components for linear traits, GIBBS1F90 is for the Bayesian analysis of linear traits and THRGIBBS1F90 is for the Bayesian analysis of threshold categorical traits [36]. For Bayesian analysis, the Gibbs sampler was run for 120 000 iterations with a burn in of 20 000 iterations, and samples from every 100^th^ sample were saved.

#### Genomic BLUP

The SNP based variance components and GEBV were estimated using GBLUP, similar to the PBLUP model (**M1**) described above. However, in GBLUP, $\boldsymbol{\mu}$ is a vector of random additive genetic polygenic effects with a distribution $\sim \mathrm{N}\left(0,\mathbf{G}{\sigma}_u^2\right)$. Here, $\mathbf{G}$ is the genomic relationship matrix, created as described by VanRaden [42]. All other parameters and details of the analysis for trait DAYS and STATUS are the same as PBLUP (**M1**).

#### SNP based BLUP method

The SNP based BLUP method used to estimate marker effects and to predict GEBV was similar to GBLUP, where *a priori* distribution of additive marker locus effects was considered to be normal [20, 42]. The model used, PSNPBLUP, combined both marker effects as well as polygenic effect (infinitesimal effect with pedigree) for all genotyped fish:

M2 $$ \boldsymbol{y}=\boldsymbol{X}\boldsymbol{\beta } +\boldsymbol{Z}\boldsymbol{a}+\boldsymbol{T}\upmu +\boldsymbol{e} $$

where $\boldsymbol{a}$ is the additive marker locus effect, $\boldsymbol{Z}$ is the incidence matrix relating to marker genotype and all other parameters are the same as PBLUP (**M1**). It was assumed that $\boldsymbol{a}$ follows *a priori* a normal distribution $\sim \mathrm{N}\left(0,\boldsymbol{I}{\sigma}_a^2\right)$, where $\boldsymbol{I}$ is an identity matrix. This model is often called ridge-regression best linear unbiased prediction with a normal distribution of marker effects [20, 42]. The trait DAYS was analyzed as a linear-mixed model in the context of Henderson’s BLUP [43] with known variances for all random effects using the keyword BLUP in the GS3 software [37]. The trait STATUS was analyzed as a threshold (probit) model assuming known variances. Random effects were estimated via Gibbs sampler using the keyword MCMCBLUP in GS3 software [37]. For both traits, the initial genetic variance ${\sigma}_{u\;}^2$ and residual variance ${\sigma}_e^2$ estimated from the model PBLUP were used to estimate additive marker variance ${\sigma}_{a\;}^2$ = ${\sigma}_{u\;}^2/2\sum {p}_i{q}_i$. Breeding values for both traits were estimated using marker effects only (without pedigree-based polygenic effect) using the model SNPBLUP:

M3 $$ \boldsymbol{y}=\boldsymbol{X}\boldsymbol{\beta } +\boldsymbol{Z}\boldsymbol{a}+\boldsymbol{e} $$

All model parameters are as described above. The BLUP was run for 10 000 iterations with convergence criteria of 10^−12^ (1d-12) and correction every 100 iterations. For MCMCBLUP, a single chain with a length of 150 000 iterations was run. The burn-in period and the thinning interval used was 50 000 and 100 iterations, respectively.

#### Bayesian estimation method: Bayes C

The Bayes C method is a mixture model for SNP effects with an assumption that there is a large group of SNPs with zero or near zero effects and a second smaller group of SNPs with larger effect [39, 44]. The Bayes C method was fitted using the same model equations as in PSNPBLUP (**M2**) and SNPBLUP (**M3**), hereafter referred to as PBAYESC and BAYESC, respectively. All model parameters are defined as above, except the elements of vector $\boldsymbol{a}$ which was calculated for each fish as:

$$ {\displaystyle \sum_{i=1}^N\left({z}_i{a}_i{\delta}_i\right)} $$

where ${z}_i$ is the genotype of *i* ^*th*^ marker, ${a}_i$ is the effect marker *i*, and ${\delta}_i$ is an indicator variable that explains if the *i* ^*th*^ marker has an effect or not. In turn, variables $\delta$ have a binomial (Bernoulli) distribution with a probability of π being 0 (marker has zero effect on the trait) and with a probability of $1-\pi$ being 1 (marker has non-zero effect on the trait). An informative beta distribution (with α and β parameters) was assumed for $\pi$ (α = 1 and β = 1, implying uniform distribution of this parameter) and inverted chi-squared distributions for the different variances ${\sigma}_a^2$, ${\sigma}_u^2$ and ${\sigma}_e^2$.

#### Bayesian estimation method: Bayesian LASSO

The Bayesian LASSO method was implemented in the context of a linear mixed model assuming an exponential prior distribution for variances of SNP marker effects [37, 45]. An alternative Bayesian implementation of the LASSO procedure [40], similar to the model equations PSNPBLUP (**M2**) and SNPBLUP (**M3**) as proposed by Legarra et al. [30], PBLASSO and BLASSO, respectively, were used. All model parameters are defined as above, except the *a priori* distributions of individual SNP effects (${a}_i$) which was calculated as:

$$ \Pr \left({a}_i\Big|{\tau}^2\right)=N\left(1,{\tau}_i^2\right)\ \mathrm{and}\; \Pr \left({\tau}_i^2\right)=\frac{\lambda^2}{2} \exp \left(-{\lambda}^2\left|{\tau}_i^2\right|\right). $$

Individual variances for each SNP (i.e., ${\tau}_i^2$) are estimated conditionally on a regularization parameter λ, which was estimated by using an *a priori* gamma distribution bounded between 0 and 10^7^. Initial value for parameter λ as ${\lambda}^2=2/{\sigma}_a^2$ was used and flat priors were assumed for ${\sigma}_a^2$, ${\sigma}_u^2$ and ${\sigma}_e^2$.

All model parameters and SNP effects estimated in a Bayesian framework (PBAYESC, BAYESC, PBLASSO and BLASSO) were estimated using the Gibbs sampling algorithm implemented in GS3 software [37]. A single chain with a length of 150 000 iterations was run. The burn-in period and the thinning interval used was 50 000 and 100 iterations, respectively.

### Genetic parameters and GEBV

The total additive genetic variance $\Big({\sigma}_{u\;}^2$) estimated in PBLUP and GBLUP models was calculated using relationship matrix $\mathbf{A}$ and $\mathbf{G}$, respectively. For both trait (DAYS and STATUS), the heritabilities were computed as:

$$ {h}^2=\frac{\sigma_u^2}{\sigma_u^2+{\sigma}_e^2}. $$

In contrast, for Bayesian models, the total additive genetic variance (${V}_{A\;}^{\prime}\Big)$ was estimated as the sum of additive marker ($2{\sigma}_{a\;}^2\pi \sum {p}_i{q}_i$) and polygenic-pedigree (${\sigma}_{u\;}^2$) based additive genetic variance; i.e., ${V}_{A\;}^{\prime }=2{\sigma}_{a\;}^2\pi \sum {p}_i{q}_i+{\sigma}_{u\;}^2$ (${\sigma}_{u\;}^2=0,$ if pedigree was not used). Here, the heritabilities were computed as:

$$ {h}^2=\frac{V_A^{\prime }}{V_A^{\prime }+{\sigma}_e^2}. $$

Additionally, in SNP based BLUP and Bayesian GS methods, the predicted GEBV were either generalized genomic breeding values (i.e., the sum of polygenic pedigree based EBV and SNP effects) or just SNP effects, depending on whether pedigree and SNP information or only SNP information was used.

### Cross validation scheme for model comparison

Predictive abilities of the different models described above (PBLUP, GBLUP, PSNPBLUP, SNPBLUP, PBAYESC, BAYESC, PBLASSO and BLASSO) were assessed through a five-fold cross validation (CV) scheme. All the fish with both phenotypes and genotypes were randomly sampled into five validation sets. The GEBV of the validation data sets were predicted one at a time where the phenotype of the validation fish (20% of the population) was masked (set to missing) and all remaining fish with phenotype and genotype (80% of the population) were used as training data. To reduce the stochastic effects, the CV analysis was replicated 10 times. Predictive ability was presented as reliability, which was estimated as:

$$ {R}_{EBV,BV}^2=\frac{R_{EBV,y}^2}{h^2}, $$

where ${R}_{EBV,\;BV}^2$ is the squared correlation between predicted (G)EBV for fish in the validation data in a given model (predicted from the training data), the recorded phenotype (*y*), and a “common” heritability (${h}^2\Big)$ of the trait which was calculated using PBLUP with full-data set and without marker information.

The Pearson’s correlation coefficients between the (G)EBV obtained by the different models was used to measure the degree of similarity between the rankings of fish. Also, for all models, the slope of regression of actual phenotype (either DAYS or STATUS) on (G)EBV were calculated and used as a measure to indicate the bias of the (G)EBV [46]. A slope of regression coefficient close to 1 indicates no bias in the model and breeding values are equal in magnitude [47]. Whereas, a slope of less than 1 or greater than 1 indicates a biased underestimation or overestimation in the (G)EBV prediction, respectively [48]. The reliability, Spearman’s rank correlation and slope of regression for each model, were reported as the average of the CV schemes used.

In addition, the effect of marker densities on the estimated reliabilities in different GS models was tested. For this, a random sample of 500, 1 K, 3 K and 20 K SNPs was used separately to predict GEBV for all GS models under the CV scheme described above. Using the lowest possible SNP density with higher or similar reliability of the 50 K SNP could help reduce genotyping costs.

## Results

### Estimated variance components with full data

Estimates of variance components with the full data set for PBLUP, GS models with combined polygenic pedigree and the 49,684 markers (GBLUP, PBAYESC and PBLASSO) and GS models with only markers (BAYESC and BLASSO) are presented for DAYS and STATUS in Table 1. The estimated residual variance (${\boldsymbol{\sigma}}_{\boldsymbol{e}\;}^2\Big)$ for DAYS was slightly lower and additive genetic variance (${\boldsymbol{V}}_{\boldsymbol{A}\;}^{\boldsymbol{\prime}}\Big)$ for both DAYS and STATUS was relatively higher in genomic models compared to PBLUP. For both traits, the estimated heritabilities were relatively higher in genomic models (${h}^2=$ 0.210±0.031 to 0.271±0.041 and 0.269±0.052 to 0.393±0.040 for DAYS and STATUS, respectively) then with PBLUP (*h* ^*2*^ = 0.185±0.038 and 0.260±0.037 for DAYS and STATUS, respectively). When comparing the PBLUP model (with $\boldsymbol{A}$ matrix) and GBLUP (with $\boldsymbol{G}$ matrix), the relative increases in estimated heritabilities were 46% for DAYS (${h}^2=$0.185±0.038 and 0.271±0.041 for PBLUP and GBLUP, respectively) and 84% for STATUS (${h}^2=$0.260±0.037 and 0.393±0.040 for PBLUP and GBLUP, respectively). Similar trends for increased estimated heritabilities compared to PBLUP were observed in Bayesian GS models for both trait DAYS (${h}^2=$0.210±0.031 to 0.231±0.034) and STATUS (${h}^2=$0.269±0.052 to 0.303±0.054). However, between the GS models, the ${h}^2$ estimated from GBLUP was higher than Bayesian models. Within the Bayesian models, the estimated heritabilities were slightly higher in the GS model with combined marker and polygenic pedigree (PBLASSO) for DAYS and in the marker-effect GS model (BLASSO) for STATUS (Table 1).

**Table 1:** Estimates of residual variance^a^ (${\boldsymbol{\sigma}}_{\boldsymbol{e}\;}^2$), additive genetic variance^b^ (${\boldsymbol{V}}_{\boldsymbol{A}\;}^{\boldsymbol{\prime}}$) and heritability^c^ (${\boldsymbol{h}}^2$) with their standard errors (±SE) for SRS resistance phenotypes DAYS and STATUS using different models^d^

Model	Trait				
	Days			Status	
	${\boldsymbol{\sigma}}_{\boldsymbol{e}\;}^2$	${\boldsymbol{V}}_{\boldsymbol{A}\;}^{\boldsymbol{\prime}}$	${\boldsymbol{h}}^2\pm \boldsymbol{S}\boldsymbol{E}$	${\boldsymbol{V}}_{\boldsymbol{A}\;}^{\boldsymbol{\prime}}$	${\boldsymbol{h}}^2\pm \boldsymbol{S}\boldsymbol{E}$
**PBLUP**	73.079	16.640	0.185 ± 0.038	0.358	0.260 ± 0.037
**GBLUP**	65.323	24.295	0.271 ± 0.041	0.661	0.393 ± 0.040
**BAYESC**	65.454	17.392	0.210 ± 0.031	0.442	0.303 ± 0.054
**PBAYESC**	65.348	19.318	0.228 ± 0.032	0.417	0.290 ± 0.053
**BLASSO**	64.963	18.037	0.217 ± 0.030	0.439	0.300 ± 0.063
**PBLASSO**	65.118	19.530	0.231 ± 0.034	0.374	0.269 ± 0.052

^a^Residual variance for binary survival STATUS was set to 1

^b, c^Total additive genetic variance ${\boldsymbol{V}}_{\boldsymbol{A}\;}^{\boldsymbol{\prime}}$: PBLUP and GBLUP was ${\boldsymbol{\sigma}}_{\boldsymbol{u}\;}^2$; BAYESC and BLASSO was $2{\sigma}_{a\;}^2\pi \sum {p}_i{q}_i$; PBAYESC and PBLASSO was $2{\sigma}_{a\;}^2\pi \sum {p}_i{q}_i+{\sigma}_{u\;}^2$

^c^Heritability ${\boldsymbol{h}}^2$ : PBLUP and GBLUP $\frac{\sigma_{u\;}^2}{\sigma_{u\;}^2+{\sigma}_{e\;}^2}$; BAYESC, BLASSO, PBAYESC and PBLASSO $\frac{V_{A\;}^{\prime }}{V_{A\;}^{\prime }+{\sigma}_{e\;}^2}$

^d^Models with pedigree: pedigree based BLUP (PBLUP), genomic BLUP (GBLUP) and Bayesian estimation methods with additive SNP effects and polygenic pedigree (PBAYESC and PBLASSO); Models with only additive SNP effects: Bayesian estimation methods (BAYESC and BLASSO)

### Correlation between predicted breeding values

The mean correlations between the predicted breeding values estimated from PBLUP (EBV) and all seven GS models (GEBV) based on five-fold cross validation are shown in Table 2. The predicted GEBV from GS models with combined polygenic pedigree and markers were highly correlated with predicted EBV (from PBLUP) for both traits DAYS (0.79 to 0.91) and STATUS (0.79 to 0.95). In addition, for both traits, the predicted GEBV from the Bayesian GS models had the highest correlation (0.84 to 0.95) followed by GBLUP (0.79). The GEBV predicted from marker-effect GS models alone had relatively lower correlation (0.76 to 0.81) with the predicted EBV for both traits. Among the GS models, the correlations between the predicted GEBV were high for both traits (0.90 to 1.0).

**Table 2:** Correlation^a^ between breeding values for SRS resistance phenotypes^b^ estimated with different models^c^ using data from 50 K SNP genotypes^d^

Model	PBLUP	GBLUP	SNPBLUP	PSNPBLUP	BAYESC	PBAYESC	BLASSO	PBLASSO
**PBLUP**		0.79	0.81	0.95	0.77	0.85	0.77	0.84
**ssGBLUP**	0.79		0.95	0.91	1.00	0.99	1.00	1.00
**BLUPSNP**	0.78	1.00		0.94	0.96	0.96	0.96	0.96
**PBLUPSNP**	0.91	0.96	0.96		0.90	0.94	0.90	0.93
**BAYESC**	0.77	1.00	1.00	0.95		0.99	1.00	0.99
**PBAYESC**	0.90	0.97	0.97	1.00	0.96		0.99	1.00
**BLASSO**	0.76	1.00	1.00	0.95	1.00	0.96		0.99
**PBLASSO**	0.91	0.97	0.96	1.00	0.96	1.00	0.96	

^a^Average Pearson correlation between breeding values estimated with different models a from five-fold cross validation scheme

^b^SRS resistance phenotypes: Survival days (DAYS) below diagonal and binary survival (STATUS) above diagonal

^c^Models with pedigree: pedigree based BLUP (PBLUP), genomic BLUP (GBLUP), marker-effects BLUP with polygenic pedigree (PSNPBLUP) and Bayesian estimation methods with marker-effects and polygenic pedigree (PBAYESC and PBLASSO); Models with only marker-effects: market-effects BLUP (SNPBLUP) and Bayesian estimation methods (BAYESC and BLASSO)

^d^The effective number of SNPs used was 49 684 from the 50 K SNP array

### Reliability and bias of different models

**Fig. 1:**
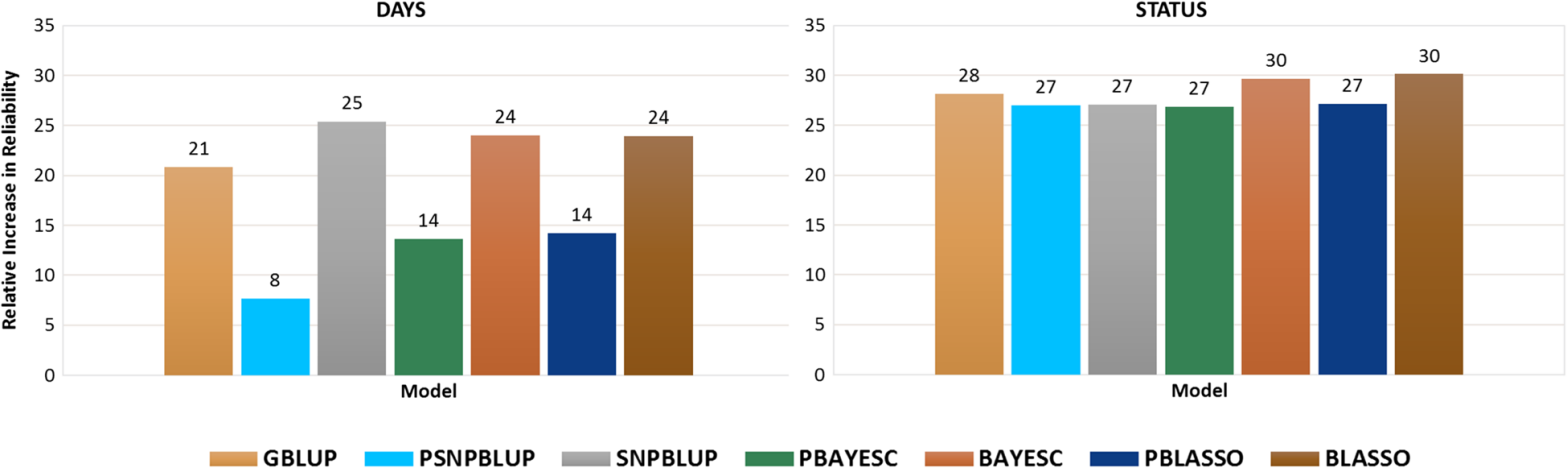
Relative increase in reliability^1^ of different genomic selection models^2^ for trait DAYS and STATUS compared with classic pedigree-based model (PBLUP). ^1^ Reliability of DAYS and STATUS using the PBLUP was 0.34 and 0.20, respectively. ^2^ Genomic selection models with pedigree and marker: genomic BLUP (GBLUP), marker-effects BLUP (PSNPBLUP) and Bayesian estimation methods (PBAYESC and PBLASSO); GS models with only marker-effects: marker-effects BLUP (SNPBLUP) and Bayesian estimation methods (BAYESC and BLASSO)

Based on the five-fold cross validation, the reliability of the PBLUP model was higher for DAYS (0.342±0.080) than for STATUS (0.201±0.038) (Table 3). Depending on whether the polygenic pedigree was used or not, the reliability of GS models ranged from 0.368±0.069 (PSNPBLUP) to 0.429±0.069 (SNPBLUP) and 0.256±0.031 (PBAYESC) to 0.262±0.026 (BLASSO) for DAYS and STATUS, respectively (Table 3). The relative increase in reliabilities for the different GS models compared with PBLUP are presented in Fig. 1 for both DAYS and STATUS. In general, all GS models outperformed the PBLUP model, but there was considerable variation between models and traits. For DAYS, the relative increase in reliability was moderate, 8 to 21% with 50 K SNP using GS models with combined polygenic pedigree and the markers (GBLUP, PSNPBLUP, PBAYESC and PBLASSO), and low (24 to 25% with 50 K SNP) for GS models with only marker-effects (SNPBLUP, BAYESC and BLASSO). In contrast, the relative increase in reliability for all GS models for STATUS were moderate and of similar magnitude (27 to 30% with 50 K).

**Table 3:** Mean reliability and bias of estimated breeding value (EBV) and genomic EBV (GEBV) for SRS survival DAYS and STATUS with their standard errors (±SE) using pedigree based and genomic models

Models^a^	Trait			
	Days		Status	
	Reliability ± *SE* ^b^	Bias ± *SE* ^c^	Reliability ± *SE*	Bias ± *SE*
**PBLUP**	0.342 ± 0.080	0.960 ± 0.146	0.201 ± 0.038	0.304 ± 0.042
**GBLUP**	0.414 ± 0.065	0.949 ± 0.097	0.256 ± 0.026	0.276 ± 0.026
**SNPBLUP**	0.429 ± 0.069	1.026 ± 0.110	0.256 ± 0.032	1.365 ± 0.096
**PSNPBLUP**	0.368 ± 0.069	0.814 ± 0.097	0.256 ± 0.039	0.798 ± 0.073
**BAYESC**	0.424 ± 0.066	0.961 ± 0.098	0.261 ± 0.026	0.287 ± 0.028
**PBAYESC**	0.389 ± 0.071	0.916 ± 0.106	0.256 ± 0.031	0.294 ± 0.029
**BLASSO**	0.424 ± 0.066	0.955 ± 0.097	0.262 ± 0.026	0.287 ± 0.026
**PBLASSO**	0.390 ± 0.072	0.937 ± 0.112	0.256 ± 0.029	0.285 ± 0.033

^a^Models with pedigree: pedigree based BLUP (PBLUP), genomic BLUP (GBLUP), marker-effects BLUP with polygenic pedigree (PSNPBLUP) and Bayesian estimation methods with marker-effects and polygenic pedigree (PBAYESC and PBLASSO); Models with only marker-effects: market-effects BLUP (SNPBLUP) and Bayesian estimation methods (BAYESC and BLASSO)

^b^The effective number of SNPs used was 49 684 from the 50 K SNP array

The bias of predicted EBV for PBLUP for DAYS (0.960±0.146) was lower than for STATUS (0.304±0.042) (Table 3), meaning DAYS deviated less from unity than STATUS. Across GS models, the bias of predicted GEBV for DAYS ranged from 0.814±0.097 to 1.026±0.110 and was similar to the PBLUP bias (Table 3). However, for STATUS, the bias of predicted GEBV across different GS models varied considerably from 0.276±0.026 (GBLUP) to 1.365±0.096 (SNPBLUP). The bias of EBV for PBLUP was 0.304±0.042 (Table 3).

### Reliability of different models at varying marker density

**Fig. 2:**
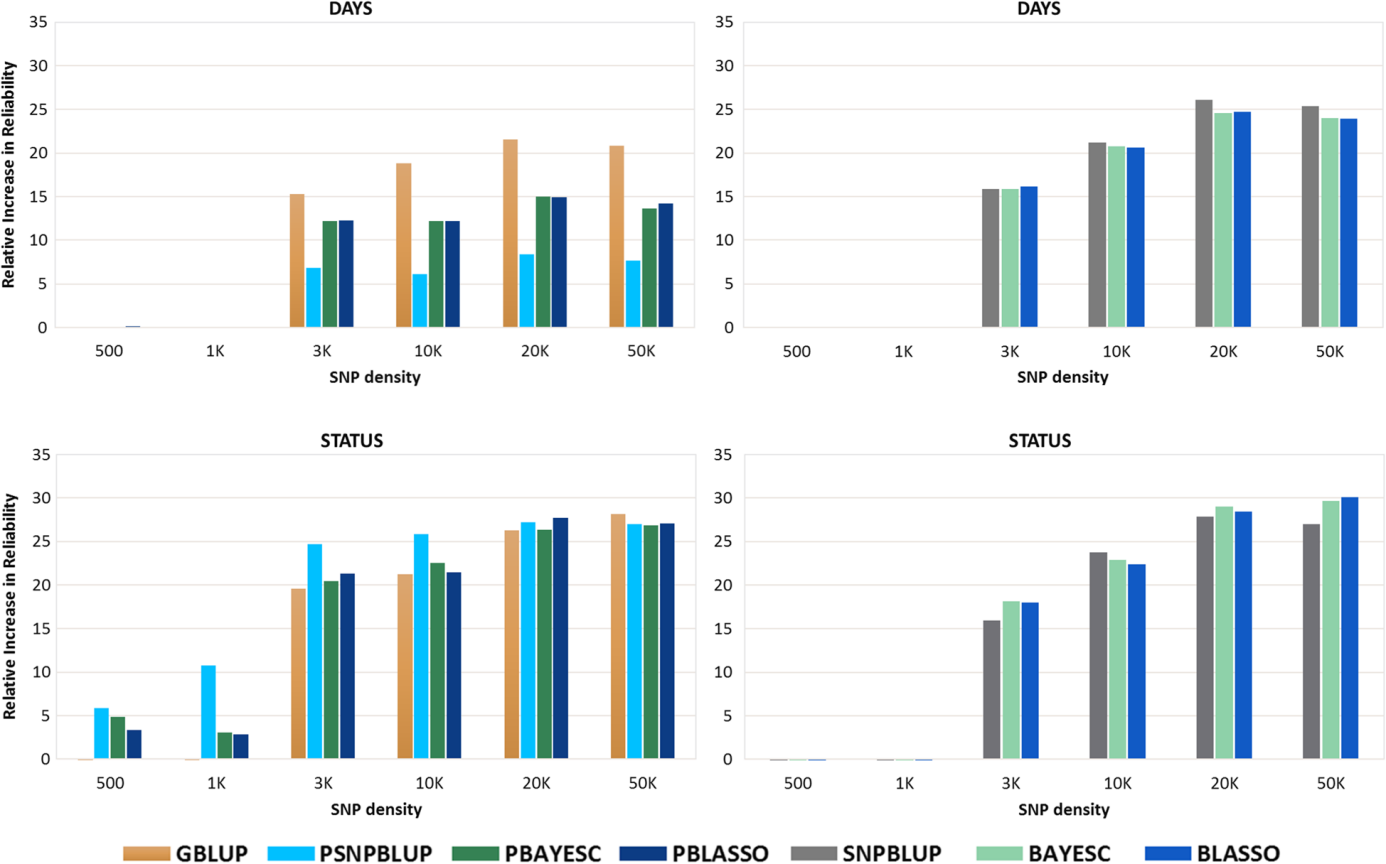
Relative increase in reliability^*a*^ of different genomic selection models^*b*^ for trait DAYS and STATUS at different SNP densities^*c*^ compared with classic pedigree-based model (PBLUP). ^*a*^ Reliability of DAYS and STATUS using the PBLUP was 0.34 and 0.20, respectively. ^*b*^ Genomic selection models with pedigree and marker: genomic BLUP (GBLUP), marker-effects BLUP (PSNPBLUP) and Bayesian estimation methods (PBAYESC and PBLASSO); GS models with only marker-effects: marker-effects BLUP (SNPBLUP) and Bayesian estimation methods (BAYESC and BLASSO). ^*c*^ SNP densities: 500, 1 000 (1 K), 3 000 (3 K), 10 000 (10 K), 20 000 (20 K) and 49 684 (50 K) SNP

The relative increase in reliability for DAYS and STATUS from different GS models was always high with higher marker densities (Fig. 2 and Additional file 2). Between different marker densities, the increase in reliability for DAYS and STATUS was 36 and 34%, respectively when going from 3 K to 20 K SNP density (Fig. 2). For both traits, the relative increase in reliability at 20 K and 50 K SNP density were of similar magnitude, suggesting that SNP density beyond 20 K would have marginal gain in selection accuracy. Nevertheless, the relative increase in predicted GEBV were superior to EBV from PBLUP even at the lowest marker density of 3 K for DAYS across GS models and marker density of 500 SNP for STATUS for GS model with pedigree and marker-effect (GBLUP, PBAYESC and PBLASSO) (Fig. 2).

## Discussion

In this study, a high density 50 K SNP array was utilized to estimate genetic parameters and to estimate predictive ability of GS models, which was then compared to traditional PBLUP for two SRS resistance traits; DAYS and STATUS in Atlantic salmon.

### Genetic parameter estimates: pedigree based and genomic heritability

The genetic variance and heritabilities estimated for this population with the PBLUP linear model for DAYS (0.185±0.038) and threshold model for STATUS (0.260±0.037) (Table 1) were consistent with the previously reported heritability estimates for SRS phenotypes, 0.18±0.03 and 0.24±0.04 for DAYS and STATUS, respectively [10, 11]. A recent study in coho salmon (*Oncorhynchus kisutch*) also reported a similar heritability estimate (0.16±0.04) for SRS resistance trait DAYS [49]. Several studies in other fish species also reported a similar range of heritability estimates for resistance to different bacterial diseases [8, 50–52]. In all these studies, genetic parameters were estimated using pedigree based relationship matrices (PBLUP as in our study).

Heritability is the central breeding program parameter used to estimate response to selection and explain the proportion of phenotypic variance due to genetics [41, 53]. The use of genomic information is expected to improve estimates of additive genetic relationships of individuals, reduce the potential confounding of additive genetic variance with residual variance, and lead to better estimates of additive genetic variance and heritability [54]. We report an increase in heritability estimates of as much as 46 and 86% for DAYS and STATUS, respectively, using genomic-relationship matrix GBLUP (Table 1). It is interesting to note that these heritability estimates are higher than estimated by Correa et al. [18] for DAYS (0.19) and STATUS (0.20) with linear and binary threshold models using genomic information from the same data set used in this study. These differences may be explained by the use of different methods to estimate heritability values from genotype data. In the present study, we used the genomic relationship matrix as described by VanRaden [42]. Correa et al. [18] used the rapid method for genome-wide pedigree-based association analysis [55, 56]. The increase in heritability estimates in our study can be attributed to better estimates of additive genetic relationship and genetic variance through the use of SNP information. The heritability estimated (from posterior means of variances) using Bayesian GS models were also higher compared to PBLUP, but lower than GBLUP (Table 1). These differences are mostly due to the fact that for Bayesian models, the total additive genetic variance was estimated as the sum of pedigree-based genetic variance (if pedigree used) and additive marker genetic variance. For instance, the BAYESC method assumes there is a large group of SNPs with zero or near zero effects and a second smaller group of SNPs with larger effect [39, 44], the BLASSO method assumes an exponential prior distribution for variances of SNP marker effects [37, 40, 45] and the GBLUP method assumes all the genotyped markers have an effect and their variance is assumed to be normal [20, 42].

For trait DAYS, the Bayesian GS models with both pedigree and SNP information (PBAYESC and PBLASSO) lead to slightly higher heritability estimates possibly because the pedigree information was useful in capturing unmarked loci that are also involved in the genetic control of this trait. In contrast, the heritability estimates were slightly lower from PBAYESC and PBLASSO for STATUS which was probably due to the scaling parameters in the model as well as the binary nature of the trait.

There is always debate about the minimum number of SNP markers required and whether causative SNPs are needed to obtain robust estimates of heritability. The low proportion of phenotypic variance in the population used in this study was previously explained to be the result of primarily polygenic control of SRS resistance with few QTLs [18]. In human studies, ~290 K common frequency SNPs explained as much as ~45% of the phenotypic variance for height [54]. Simulation studies have shown that a few thousand markers are enough to accurately estimate heritability [57], and inclusion of causative SNPs has little effect on prediction accuracy [58]. Moreover, it is not necessary to know causative SNPs or SNPs that are closely linked to the causative SNPs to obtain reliable estimates of heritability [59]. Therefore, the heritabilities estimated using 49,684 SNPs in the current study can safely be considered reliable.

### Ranking of EBV and GEBV

The GEBV predicted from the genomic relationship based GBLUP and marker-effect based GS models (SNPBLUP, BAYESC and BLASSO) for both SRS resistance traits were moderately correlated (0.76 to 0.81) with the predicted EBV (Table 2). These results suggest that predicted EBV (with $\boldsymbol{A}$ matrix) and GEBV (with $\boldsymbol{G}$ matrix or marker-effect alone) are somewhat different predictors of genetic merit of fish, for these two SRS traits, in this population. However, the predicted GEBV for both traits with the combined pedigree and marker-effect GS models (PSNPBLUP, PBAYESC and PBLASSO) showed high correlation (0.90 to 0.95) with the predicted EBV. In contrast, for resistance to bacterial cold water disease (BCWD) a low correlation (~0.60) between predicted EBV from PBLUP and GEBV from marker-effect based Bayesian models was reported [50]. The predicted GEBV between all GS models were highly correlated (Table 2) which is in agreement with results from Vallejo et al. [50]. These high correlations indicate similar ranking of full-families between PBLUP and GS methods, and within different GS methods.

### Reliability of PBLUP and GS models

The accuracy of breeding values estimated in terms of reliability for DAYS (0.342±0.080) was similar to the previously reported reliabilities for fillet color (0.36) and lice resistance (0.34) in Atlantic salmon using the PBLUP model [9]. However, the trait STATUS was bit lower (0.201±0.038) (Table 3). The reliabilities of EBV and GBEV were relatively higher for DAYS compared to STATUS, possibly due to the better fit of linear trait DAYS with the linear models than the binary trait STATUS with the threshold models (Table 3). This is in agreement with the predictive abilities (the correlation between mid-parent EBV or GEBV and the mean progeny phenotype) reported for BCWD which were comparatively higher for DAYS (0.50 for EBV and 0.37 to 0.49 for EBV and GEBV, respectively) than STATUS (0.41 and 0.26 to 0.46 for EBV and GEBV, respectively) [50].

All GS models outperformed the PBLUP model with respect to estimated reliabilities for both the traits (Table 3 and Fig. 1). In other simulation studies, different GS methods also showed significantly higher accuracy compared to PBLUP in the typical half/full-sibling family structure of a salmon breeding program [22, 25, 60]. A study by Ødegård et al. [9] showed an improvement in accuracies (reliabilities) of 32 to 51% for sea lice resistance and, up to 22% for fillet color. For traits such as weight and length in juvenile salmon as much as a 20% improvement of accuracies can be obtained by applying GBLUP compared to PBLUP [26].

It was interesting to note that reliability of GEBV estimated from combined pedigree and marker effect based GS models (PSNPBLUP, PBAYESC and PBLASSO) were lower than the models with only marker-effects for the trait DAYS (SNPBLUP, BAYESC and BLASSO) (Table 3 and Fig. 1). The GEBV from combined pedigree and marker-effect GS models were expressed as “generalized” GEBV, i.e., the sum of “polygenic” and the SNP effects [37]. Whereas, the GEBV from marker-effect GS models were just the sum of SNP effects which showed high correlation with GEBV from GBLUP (Table 2). Therefore, the presence of the polygenic EBV component in the GEBV (from PSNPBLUP, PBAYESC and PBLASSO) showed high correlation with EBV from PBLUP (Table 2) and reliabilities were closer to that of EBV. Interestingly, GS models with pedigree and marker-effects for DAYS showed reliabilities closer to that of PBLUP, possibly because the GEBV predicted in these models had over-representation of polygenic EBV (Table 3 and Fig. 1).

The reliabilities of predicted GEBV from different GS models were very close and the differences between GS models were negligible for both traits (Table 3 and Fig. 1). On the contrary, Vallejo et.al. [50] reported a relatively higher predictive ability of GBLUP compared to Bayesian GS methods using a different GS design and a much smaller number of genotyped samples. Recently, we have shown that, resistance to SRS is primarily controlled by polygenic inheritance (i.e., many loci explaining very small effects of the trait) [18]. The GEBV predicted with GBLUP utilizes a more accurate genetic relationship calculated from shared SNP genotype data and pedigree information rather than just the expected average relationship used in PBLUP [61–63]. Therefore, GBLUP may perform better when we have close family relationships in the data as in an aquaculture breeding program. The SNP based BLUP (PSNPBLUP and SNPBLUP) GS models do not use a genomic relationship matrix and fits SNP information as random effects. The Bayesian variable selection GS models usually fit markers with only moderate to large effect [64], and are time consuming for routine genetic evaluations. Similar to our findings, it has been shown that GBLUP and Bayesian methods (Bayes B) achieve very similar accuracies in dairy cattle data GS analysis for most traits [19, 65]. Therefore, considering the high correlation of GEBV between GBLUP and Bayesian GS methods and negligible differences between reliabilities, the GBLUP method may be an attractive approach for the routine application of GS to select for SRS resistance in Atlantic salmon.

### Effect of marker density on reliability

There is always a debate around the effect of marker density on GS prediction accuracy. The use of a low marker density panel may represent a cost-effective approach for GS prediction especially for aquaculture where thousands of potential breeders need to be genotyped. However, high density marker panels are expected to be more accurate and whole genome sequencing data or targeted causative variants genotyping are expected to give higher accuracies [66]. The choice of number of markers for accurate GEBV prediction also depends on the LD between the markers and the QTLs [12]. The use of a low density SNP panel with low LD between the markers may result in inaccurate prediction of genetic values for human height as suggested by Yang et.al. [54]. The Atlantic salmon reference assembly genome is up to 2.97 gigabases [67] with roughly 2970 centiMorgans (cM). The total SNP data set with 49,684 SNPs analyzed in the present study represented an average genome coverage of ~16.70 SNPs per cM. A simulation study by Vela-Avitúa et al. [60] showed that an identical-by-descent relationship based GS applied to a typical aquaculture breeding program across traits with different heritabilities (h2 ~0.1, 0.3 and 0.8) even using sparse markers (10–20 SNPs/M) showed higher prediction accuracies than PBLUP.

In our study, a marker density less than 3 K gave considerably lower reliability of GEBV, which was likely due to insufficient LD between the markers caused by the large distance between the randomly selected markers (Fig. 2). In addition, there was considerable gain in reliability observed from a marker density of 3 K to 20 K. As discussed by Ødegård et.al. [9], the salmon breeding population used in this study historically originated from admixture of several distinct wild strains with expected long-range LD. This might explain the increased reliability of GS models with sparse marker densities as low as 3 K and up to 20 K.

The choice of exact marker density for genotyping a large number of potential breeders would largely depend on the added cost of genotyping and the economic benefit obtained by the extra gain in accuracy of the trait under selection. The cost-benefit is also likely to be most favorable for traits that cannot be measured on potential breeders (e.g., disease resistance, meat quality traits) and traits with high economic value (e.g., SRS in Chilean salmon industry.). Also, the marker density of 20 K gave a reliability close to that of highest marker density (50 K) showing that the LD between markers at 20 K and 50 K are similar and no additional gain would be obtained using a marker density beyond 20 K (Fig. 2). This is in agreement with the findings of Ødegård et.al. [9] that little increase in accuracy was observed with a marker density above 22 K for fillet color or lice resistance in a commercial salmon population.

## Conclusions

Our results show that different genomic selection models applying a 50 K SNP array showed higher accuracy of breeding value prediction in terms of reliability than the model using only pedigree based relationship, PBLUP, for both DAYS and STATUS with an improvement of approximately 25 and 29%, respectively. In the current population, ~20,000 high quality informative SNPs was enough to achieve a similar increase in prediction accuracy. A marker density as low as 3 K and 500 SNP performed better than PBLUP for DAYS and STATUS, respectively. Therefore, using a lower SNP density (e.g., 20 K SNP) or the combination of low SNP density (e.g., 500 SNP) and an imputation strategy may help reduce genotyping cost without compromising the gain in reliability. We are currently working on an imputation strategy to explore the possibility of reducing the genotyping cost. The BLUP model which uses genomic relationship calculated from pedigree as well as SNP information (GBLUP) performed similar to the SNP based BLUP GS models and Bayesian variable selection GS models (Bayes C and Bayesian Lasso). The relative advantage of using SNP data to improve disease resistance depends on the cost of the disease challenge test to collect SRS phenotypes, genotyping thousands of training candidates (candidates with phenotype) and validation (potential breeders without phenotypes) which are expensive. The added economic impact of the extra improvement in SRS resistance needs to be evaluated carefully.

## Additional files

Additional file 1: Figure S1. The Kaplan-Meier curves of the survival function was plotted for the test period to show the cumulative mortality across the challenge. (PDF 5 kb)
Additional file 2: Reliability from five-fold cross validation steps for PBLUP and different GS models for DAYS and STATUS at different marker densities; Mean reliability table for BLUP and all GS models for DAYS and STATUS at different marker densities and corresponding plots; Increase in reliability (in percentage) for all GS models for DAYS and STATUS at different marker densities compared to PBLUP and corresponding plots. (XLSX 74 kb)

## Abbreviations

BAYESC: Bayes C with marker-effects
BLASSO: Bayesian LASSO with marker-effects
cM: centiMorgans
EBV: Estimated breeding values
GBLUP: Genomic BLUP
GEBV: Genomic EBV
GS: Genomic selection
GWAS: Genome-wide association study
LD: Linkage disequilibrium
MAS: Marker Assisted Selection
PBAYESC: Bayes C with marker-effects and polygenic pedigree
PBLASSO: Bayesian LASSO with marker-effects and polygenic pedigree
PBLUP: Pedigree based best linear unbiased pediction
ppt: Parts per thousand
PSNPBLUP: Marker-effects BLUP with polygenic pedigree
QTL: Quantitative trait loci
SD: Standard deviation
SNP: Single nucleotide polymorphisms
SNPBLUP: Marker-effects BLUP
SRS: Salmon rickettsial syndrome
