# Supplementary figures and images for: Genomic predictions can accelerate selection for resistance against Piscirickettsia salmonis in Atlantic salmon (Salmo salar)
Source: BMC Genomics. 2017 Jan 31;18:121. doi: 10.1186/s12864-017-3487-y (PMC5282740; doi:10.1186/s12864-017-3487-y)

**Kaplan–Meier Curve**

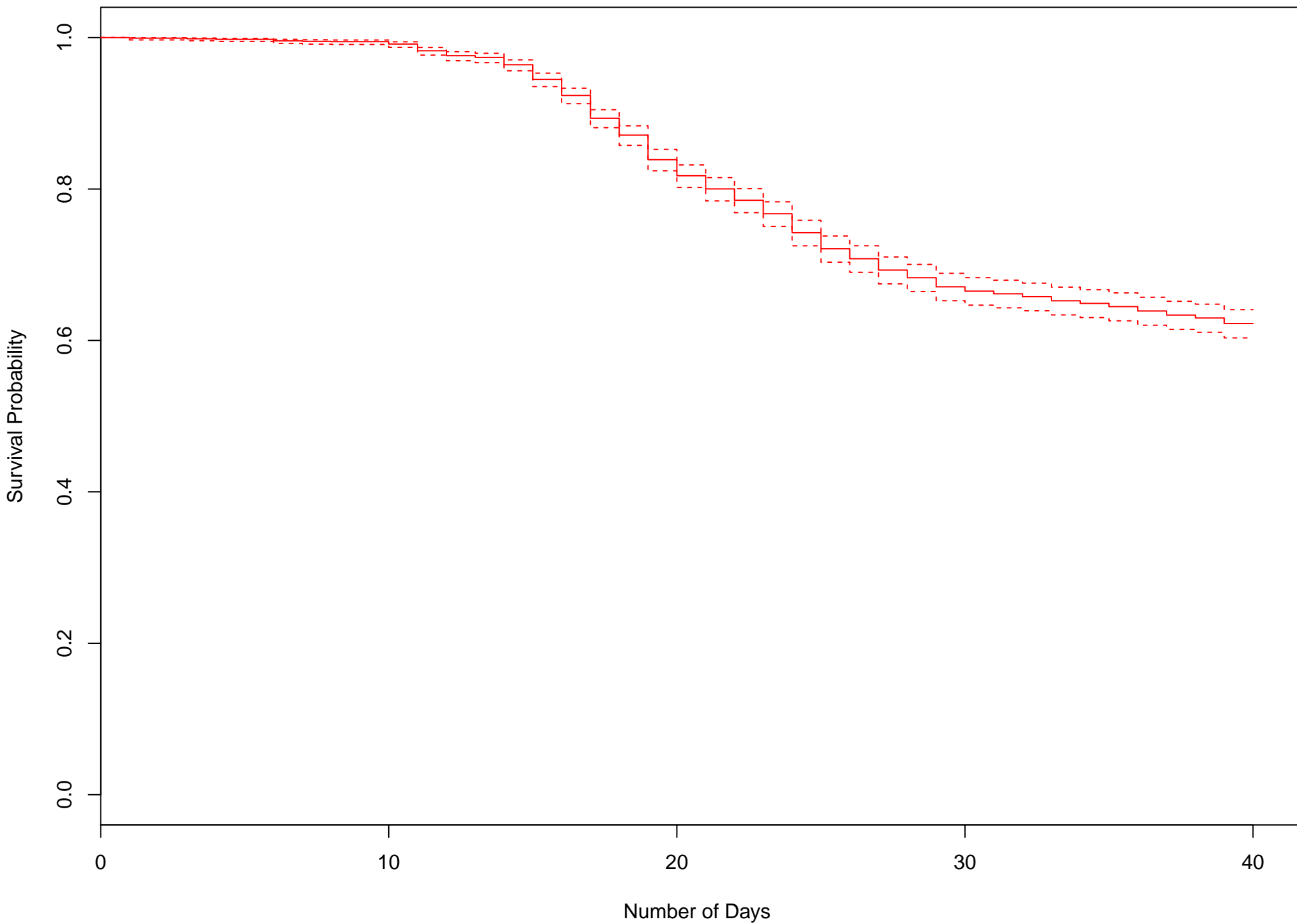

Supplement: Additional file 1: Figure S1. — The Kaplan-Meier curves of the survival function was plotted for the test period to show the cumulative mortality across the challenge. (PDF 5 kb) [file 12864_2017_3487_MOESM1_ESM.pdf]
